# EGR1 inhibits clear cell renal cell carcinoma proliferation and metastasis via the MAPK15 pathway

**DOI:** 10.32604/or.2024.056039

**Published:** 2025-01-16

**Authors:** NAIXIONG PENG, YUEFENG CAI, DONG CHEN, LING DENG, ZEJIAN ZHANG, WEI LI

**Affiliations:** Department of Urology, Shenzhen Longhua District Central Hospital, Shenzhen, 518110, China

**Keywords:** Early growth response factor 1 (EGR1), Migration, Proliferation, Mitogen-activated protein kinase 15 (MAPK15), Clear cell renal carcinoma (ccRCC)

## Abstract

**Background:**

Clear cell renal carcinoma (ccRCC), the leading histological subtype of RCC, lacks any targeted therapy options. Although some studies have shown that early growth response factor 1 (EGR1) has a significant role in cancer development and progression, its role and underlying mechanisms in ccRCC remain poorly understood.

**Methods:**

The Cancer Genome Atlas (TCGA) database was utilized to examine the expression of EGR1 in ccRCC. The expression of EGR1 in 55 ccRCC tissues was evaluated using immunohistochemistry. The link between EGR1 expression and clinicopathological variables was examined through an analysis. Gain-of-function assays were employed to investigate EGR1’s biological functions in ccRCC cells, involving proliferation, colony formation, invasion assays, and tumorigenesis in nude mice. In order to assess the protein expression of mitogen-activated protein kinase 15 (MAPK15), E-cadherin, matrix metalloproteinase-9/-2 (MMP-9 and MMP-2), Western blot technique was applied.

**Results:**

The results revealed a decrease in EGR1 expression in ccRCC tissues. This decrease was strongly linked to TNM stage, lymphatic metastasis, tumor size, histological grade, and unfavorable prognosis in ccRCC patients. It has been demonstrated that overexpressing EGR1 inhibits the growth of xenograft tumors *in vivo* and inhibits cell colony formation, motility, and invasion *in vitro*. Furthermore, EGR1 can prevent the development and movement of ccRCC cells by controlling the expression of MMP-2, MMP-9, E-cadherin, and MAPK15.

**Conclusions:**

The EGR1/MAPK15 axis may represent a promising target for drug development, with EGR1 serving as a possible target for ccRCC therapy.

## Introduction

Renal cell carcinoma (RCC), which arises from the renal epithelium, is the predominant form of kidney cancer, and its incidence is on the rise [1]. RCC can be classified into several histological subtypes based on where it originates in the renal tubules. Clear cell renal cell carcinoma (ccRCC) is the most malignant subtype and the leading cause of cancer-related mortality [2]. The treatment of metastatic ccRCC has advanced significantly, with targeted drugs like sorafenib suppressing vascular endothelial growth factor (VEGF) and its receptor (VEGFR). However, the average 5-year survival rate for metastatic cases is only 10% [3,4].

Additionally, because there are rarely any early symptoms, many ccRCCs are discovered at an advanced stage. The unavailability of practical tumor markers in clinical practice makes diagnosing tumors difficult [5]. Therefore, studying the molecular characteristics of ccRCC and mining important biomarkers as potential targets for early diagnosis, monitoring, and treatment significantly impact the prevention and management of renal clear cell carcinoma.

The early growth response (EGR) gene family consists of four transcription factors containing zinc finger domains: EGR1, EGR2, EGR3, and EGR4 [6]. The EGR1 is the most crucial member of the EGR family [7,8]. The EGR1 transcription factor can bind to specific target genes and control their transcription because it has a zinc finger domain. Prior research has shown that EGR1 primarily functions during immunological responses, fibrosis, and tissue damage [9,10]. Recent findings reveal that EGR1 plays different regulatory roles in various tumor tissues, accelerating tumor development and expansion by enhancing cell proliferation, angiogenesis, and invasion, while also demonstrating cancer-inhibiting properties and inducing apoptosis in tumor cells [7]. Several researches suggest that EGR1 is implicated in developing resistance to cancer treatments [11,12]. As a transcription factor, EGR1 influences gene expression by either promoting or inhibiting the transcription of its target genes. Several tumor suppressor genes, including transforming growth factor β1, phosphatase and tensin homolog (PTEN), and tumor suppressor gene p53, are directly regulated by EGR1 [13–15]. Thus, understanding the function and molecular regulation of EGR1 in tumors holds significant theoretical and clinical importance.

Despite previous research showing a significant reduction of EGR1 protein in ccRCC tissues, the exact role of EGR1 and the molecular mechanism by which it regulates ccRCC progression is still unknown [16]. The present investigation used the TCGA database, clinical samples, and cell function tests to explore the function of EGR1 in ccRCC and its initial molecular regulation mechanism.

## Materials and Methods

### Data retrieval

Firstly, the TCGA (https://tcga-data.nci.nih.gov/) (accessed on 11 October 2024) database was retrieved to analyze the expression profiles of EGR family members, including EGR1, EGR2, EGR3, and EGR4, in ccRCC transcriptome data. The parameters were log_2_Fold change > 1 and *p* < 0.01. Using the TCGA database, the clinical significance of EGR1 in ccRCC was evaluated, such as its relationship with survival rates.

### Data analysis and immunohistochemistry (IHC) staining

The ccRCC genes found to be differentially expressed were from the TCGA database. Shanghai Outdo Biotech Co., Ltd. (Shanghai, China) also supplied a tissue array with follow-up data comprising 55 pairs of ccRCC samples and paired neighboring normal renal tissues.

IHC staining was performed following the previously published protocol [17]. In brief, the sections were exposed to an anti-EGR1 antibody (1:100 dilution, Abcam, ab6054, Abcam, UK) for an extended period at a temperature of 4°C. Afterwards, the sections were seen using a 3, 3-diaminobenzidine (DAB) kit (T15132, Shanghai Shangbao Biotechnology Co., Ltd., Shanghai, China). Two pathologists, blinded to the origins of the samples and patient outcomes, assessed all IHC samples independently. Two middle-ranking doctors in the pathology department reviewed the histochemical section separately. The reading content includes the expression localization of EGR1.

The overall score was determined by multiplying the degree of staining (absence of staining = 0; weak staining = 1 point; moderate staining = 2 points; intense staining = 3 points) with the percentage of cells showing positive staining (3 points for >75%; 2 points for 50%~75%; 1 point for 25%~50%; 0 points for <25%). Expressions with scores over 4 were categorized as high, while expressions with 4 or less were classified as low.

### Transfection and cell culture assay

As previously pointed out, the human ccRCC cell lines (A498, 796-P, 786-O, and ACHN) and normal proximal tubule epithelial cell line HK-2 were employed [17]. 796-P and 786-O were cultured on Dulbecco’s Modified Eagle Medium (12491015, DMEM, Gibco, Carlsbad, CA, USA). A498, HK-2 and ACHN were cultured in RPMI 1640 (C0893, Beyotime Biotech, Shanghai, China) containing 10% fetal bovine serum (FBS, 12103, Sigma-Aldrich, St. Louis, MO, USA), 100 U/mL penicillin, and 100 ug/mL streptomycin (C0222, Beyotime Biotech, China) in an incubator with 5% CO_2_. After testing, it was determined that none of the cell lines had Mycoplasma. According to the EGR1 sequence (Gene ID: 1958), pcDNA3.1-EGR1 (OV-EGR1) and control vector (Control-EV) were constructed by Genechem (Shanghai, China). Each well of the 6-well plates received 2 × 10^5^ cells for plating. Once the cell culture achieved 80% confluence, it was infected with lentiviruses overexpressing EGR1 at a multiplicity of infection (MOI = 10) following the instructions outlined by the manufacturer. Following 48 h, the effectiveness of transfection was assessed using Western blot (WB) and quantitative RT-PCR (qRT-PCR) assays.

### RNA extraction and qRT-PCR assays

TRIzol reagent (15596026, Thermo Scientific, Waltham, MA, USA) was used to prepare cells for total RNA isolation following the instructions outlined by the manufacturer. The RNA quantity and purity were determined by measuring absorbance at 260 and 280 nm using the NanoDrop 2000 (Thermo Scientific, MA, USA). Following manufacturer directions, the SuperScript III First-Strand Synthesis Kit (18080-051, Thermo Scientific, MA, USA) synthesized cDNA. The mRNA levels were measured in real-time using the Bio-Rad iQ5 Real-Time PCR System(170-9780, Bio-Rad, Hercules, CA, USA) and GAPDH as the reference gene. The 2^−ΔΔCt^ approach was applied to determine the amounts of target mRNA, with experiments conducted in triplicate [18]. Primer sequences were based on those reported in a previous study [15]. The primer sequences are given below:

EGR1 forward: ′-CTGCGACATCTGTGGAAGAAA-3′, EGR1 reverse: 5′-TGTCTGCTTTCTTGTCCTTCTG-3′, GAPDH forward: 5′-GGAGCGAGATCCCTCCAAAAT-3′; GAPDH reverse: 5′-GGCTGTTGTCATACTTCTCATGG-3′.

### Cell proliferation assay

EGR1’s effect on ccRCC cell proliferation was measured with the MTT assay, as detailed in the previously reported protocol [17]. In summary, after being digested, 200 μL of transfected ccRCC cells were added to each well of 96-well plates, resulting in a density of 2.0 × 10^3^ cells per well. Then, 10 μL of 5 mg/mL of MTT solution (No. 23934, Sigma-Aldrich, MO, USA) was added to the cells after culture at different times (1 day, 2 day, 3 day and 4 day). To dissolve the crystals, 100 μL of DMSO was substituted for the medium. The plate was subsequently agitated for 10 min. After that, the absorbance was determined using a microtiter plate reader (BIO-TEK Instruments, Winooski, VT, USA) at a wavelength of 590 nm.

### Colony formation assay

ccRCC cells that had been transfected were trypsinized and dispersed into 6-well plates at 600 cells per well density and a volume of 2 mL per well. The culture was cultured in a constant temperature medium of 37°C and 5% CO_2_, and every three days, the medium was changed. After 14 days, fix cells for 60 min using 1 mL of 4% paraformaldehyde (P0099, Beyotime Biotech, China) at a temperature of 4°C, wash with Phosphate-Buffered Saline (1 × PBS) (C0221A, Beyotime Biotech, China), and stain with 0.1% crystal violet for 2 min. Photographs of the 6-well plates were taken, and visible colonies with more than 50 cells were examined.

### Wound-healing assay

The ccRCC cells that had undergone transfection were enzymatically digested and 5 × 10^4^ cells were placed into 6-well plates. A 200 μL micropipette tip created a straight wound in the completely confluent monolayer of cells after a 12-h growth period (in RPMI 1640 containing 10% FBS). The area was then washed with PBS to eliminate any cellular waste. Cell migration was identified after a 24-h incubation period using a 40 × magnification microscope (CK40, Olympus Corporation, Tokyo, Japan). The inhibitory impact was seen upon normalization, with the quantity for the control group set as 100%.

### Cell migration and invasion assays

To assess the migration and invasion capabilities of transfected ccRCC cells, the cells were digested and seeded into a transwell insert designed for a 24-well plate featuring an 8 μm pore size (Corning, NY, USA). For migration assays, 2.5 × 10^4^ transfected ccRCC cells were introduced into the transwell insert. The invasion tests were conducted using a similar procedure, except the upper chambers of the 24-well inserts were covered with Matrigel (356225, BD Biosciences, Bedford, MA, USA) at a 200 mg/mL concentration. The cells relocated to the lower chamber were treated with a 0.1% solution of crystal violet (C0121, Beyotime Biotech, China) to produce a stain after being incubated for 48 h at 37°C. A light microscope (CK40; Olympus Corporation, Tokyo, Japan) was used to examine the stained cells at a magnification of 200×. Images were taken, and cell counting was performed randomly across five fields, with the results averaged for analysis.

### Western blot

Proteins were extracted following a previously established protocol [17]. The protein concentration was determined using the BCA Protein Assay Kit (23225, Pierce, Rockford, IL, USA). The protein was evenly distributed onto 12% SDS-PAGE gels to be separated and transferred to polyvinylidene difluoride (PVDF) membranes (IPVH00010, Millipore Co., Billerica, MA, USA) by electroblotting, with equal amounts of protein loaded. For 1 h at room temperature (RT), 5% non-fat dry milk was used to block the membranes. Primary antibodies were then applied overnight at a temperature of 4°C, including anti-EGR1 (ab6054, 1:1000, Abcam, Cambridge, UK), anti-E-cadherin (EP700Y, 1:800, Abcam, Cambridge, UK), anti-MMP-9 (JA80-73, 1:1000, Thermo Fisher Scientific, USA), anti-MMP-2 (#4022, 1:1000, CST, USA), and anti-GAPDH (ab8245, 1:1000, Abcam, Cambridge, UK). On the following day, the membranes were exposed to secondary antibodies (AS014,Abclonal, Wuhan, China) linked to HRP at a dilution of 1:3000. The incubation took place at RT for 1 h. Enhanced chemiluminescence (ECL) (ECL-F-100, Yanxi Biotechnology Co., Ltd., Shanghai, China) reagents were used to detect protein bands, with beta-actin as the internal reference, and then analyzed using Image J software (version 1.8.0, National Institute of Health, Bethesda, MD, USA).

### In vivo xenograft assay

Guangdong Medical University approved all *in vivo* investigations, which complied with the procedures established for the Care and Use of Laboratory Animals (Approval No. GDY2102011). A total of ten 4-week-old male BALB/c nude mice were obtained from the Guangdong Medical Laboratory Animal Center in Guangzhou, China. The mice had a weight ranging from 18 to 20 grams and were housed under specific pathogen-free conditions following the ARRIVE guidelines.

The mice were randomly divided into 2 groups (n = 5) using a random number table method. The transfected ccRCC cells (786-O) were digested, and 10^5^ cells suspended in 100 μL of PBS were injected under the skin of nude mice. Tumor volumes were calculated 12 days afterwards with the formula volume (mm^3^) = L × W^2^ × 0.5, where L represents the length, and W represents the width. After 30 days, nude mice were killed using 100% CO_2_ for 5 min. Then the tumor was surgically removed and weighed.

### Statistical analysis

Statistical analysis of the two groups was conducted using GraphPad Prism 9.0 software (La Jolla, USA). Quantitative data are presented as mean ± standard deviation (SD), and comparisons between groups were made using the student’s *t*-test. Categorical data are expressed as counts (n), and comparisons between groups were analyzed using the chi-square test. The survival curves were plotted using the Kaplan-Meier method. To investigate differences across groups, the log-rank test was used. The multivariate analysis found the independent prognostic factors through the Cox proportional hazard model. The statistical significance was indicated by a *p*-value < 0.05.

## Results

### Decreased EGR1 levels in ccRCC

**Figure 1:**
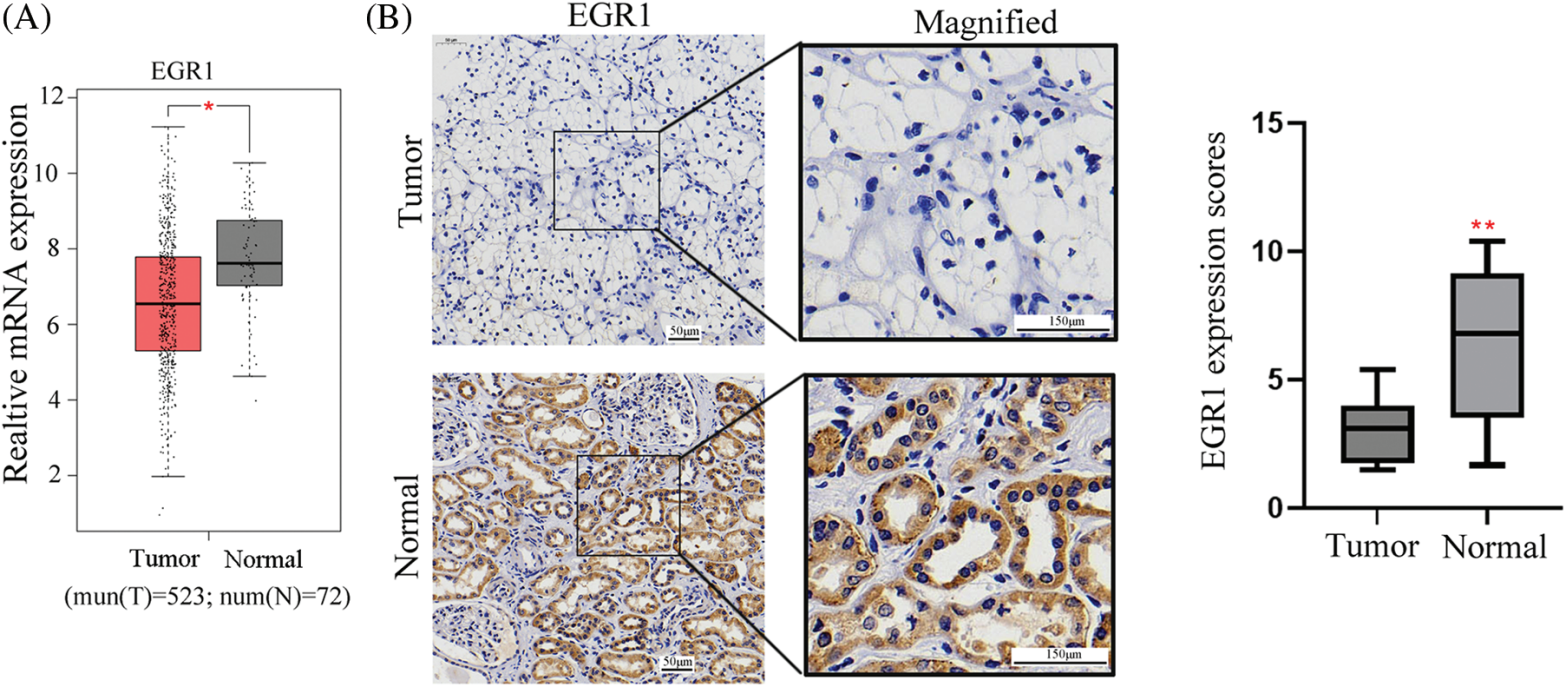
EGR1 expression in ccRCC tissues. The figure shows (A) the EGR1 mRNA expression in ccRCC tissues and para-cancerous tissues analyzed in TCGA data and (B) the IHC of EGR1 expression in ccRCC tissues, n = 55. T: tumor, N: Normal. *p < 0.05, **p < 0.01.

**Figure A1:**
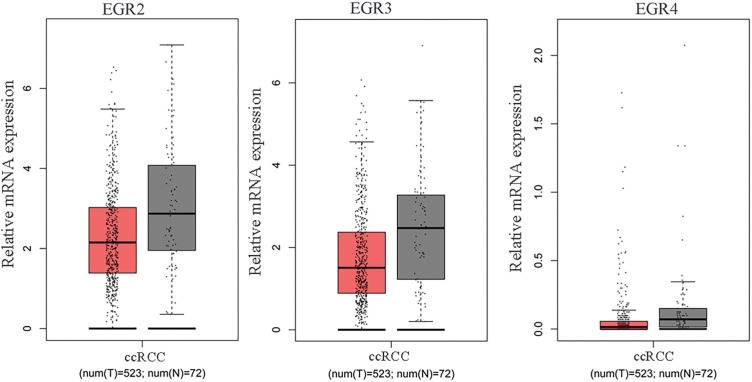
The mRNA expression levels of EGR2, EGR3 and EGR4 in ccRCC tissues and adjacent tissues (normal) were analyzed utilizing published ccRCC transcriptome data accessed from the TCGA database, T: tumor, N: normal.

Firstly, the TCGA database was employed to evaluate the expression profiles of EGR family members, including EGR1, EGR2, EGR3, and EGR4, in ccRCC transcriptome data. The finding revealed that compared with the paracancer (normal) tissues, only EGR1 was significantly reduced in ccRCC tissues (Figs. 1A, A1) (*p* < 0.05). The ccRCC tissues’ EGR1 levels were much lower, as shown by the IHC staining (Fig. 1B) (*p* < 0.01). These findings demonstrated that the EGR1 protein was substantially reduced in ccRCC tissues compared to adjacent normal renal tissues.

A total of 55 ccRCC patients were divided into two groups based on the immunohistochemistry EGR1 scores: the high EGR1 expression group (n = 16) (scores >4 are considered a high expression) and the low EGR1 expression group (n = 39) (scores <4 are considered a low expression). The current investigation assessed the association between the clinicopathological characteristics of patients with ccRCC and EGR1 expression. The analysis revealed that EGR1 expression was unrelated to age or sex (*p* > 0.05). Nevertheless, EGR1 expression is associated with histological grade, lymphatic metastasis, tumor size, and TNM stage in patients (*p* < 0.05) (Table 1). In the univariate survival analysis, it was found that TNM stage, tumor size, histological grade, and EGR1 expression are factors influencing the prognosis of ccRCC patients (*p* < 0.05) (Table 2). Further multivariate analysis revealed that TNM stage and EGR1 expression are independent risk factors affecting the prognosis of ccRCC (*p* < 0.05) (Table 2).

**Table 1:** Correlation between EGR1 protein expression and clinicopathological features of ccRCC patients

Variable	n	High expression (n = 16)	Low expression (n = 39)	χ^2^	*p
Age (years)				0.0346	0.8524
≤60	32	9	23		
>60	23	7	16		
Sex				0.1084	0.7420
Male	36	11	25		
Female	19	5	14		
TNM stage				9.025	**0.0027**
I–II	24	12	12		
III–IV	31	4	27		
Tumor size (cm)				4.760	**0.0291**
≤4	22	10	12		
>4	33	6	27		
Lymphatic metastasis				5.786	**0.0162**
Yes	31	5	26		
No	24	11	13		
Histological grade				6.716	**0.0348**
1–2	20	10	10		
3	25	4	21		
4	10	2	8		

Note: *Chi square test. The meaning of the bold values is highlighted and less than 0.05.

**Table 2:** COX univariate and multivariate analysis of prognosis in ccRCC patients

Variable	Single factor analysis			Multiple factor analysis		
	HR	95%CI	p*	HR	95%CI	*p* ^#^
Age (years)						
≤60	0.524	0.155–1.770	0.298			
>60	1.000					
Sex						
Male	1.519	0.649–3.556	0.336			
Female	1.000					
TNM stage						
I–II	0.041	0.006–0.307	**0.002**	0.114	0.014–0.914	**0.041**
III–IV	1.000					
Tumor size (cm)						
≤4	0.194	0.057–0.657	**0.008**	0.740	0.194–2.830	0.660
>4	1.000					
Lymphatic metastasis						
Yes	1.7704	0.736–3.946	0.213			
No	1.000					
Histological grade						
1–2	0.067	0.008–0.560	**0.013**	0.233	0.027–1.993	0.183
3	1.219	0.473–3.146	0.682	1.307	0.4677–3.658	0.610
4	1.000					
EGR1 expression						
High	0.080	0.019–0.345	**0.001**	0.177	0.037–0.844	**0.030**
Low	1.000					

Note: *Log-rank test, ^#^Cox-regression model, The meaning of the bold values is highlighted and less than 0.05.

**Figure 2:**
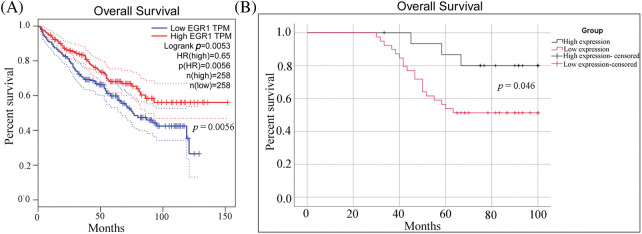
The OS curve for ccRCC patients with different levels of EGR1 expression. (A) The OS survival curves of EGR1 in the TCGA database. (B) The OS survival curves of EGR1 in ccRCC patients according to EGR1 expression based on IHC staining.

Additionally, the Kaplan-Meier survival curves based on the TCGA database showed that participants with elevated EGR1 expression reported a higher OS rate than patients with lower EGR1 expression (Fig. 2A). The survival curves for both patient groups were produced through the Kaplan-Meier technique. These findings suggested that in ccRCC, reduced EGR1 expression was substantially linked to a lower OS rate (*p* = 0.046) (Fig. 2B).

### Overexpression of EGR1 inhibited the growth of ccRCC

**Figure 3:**
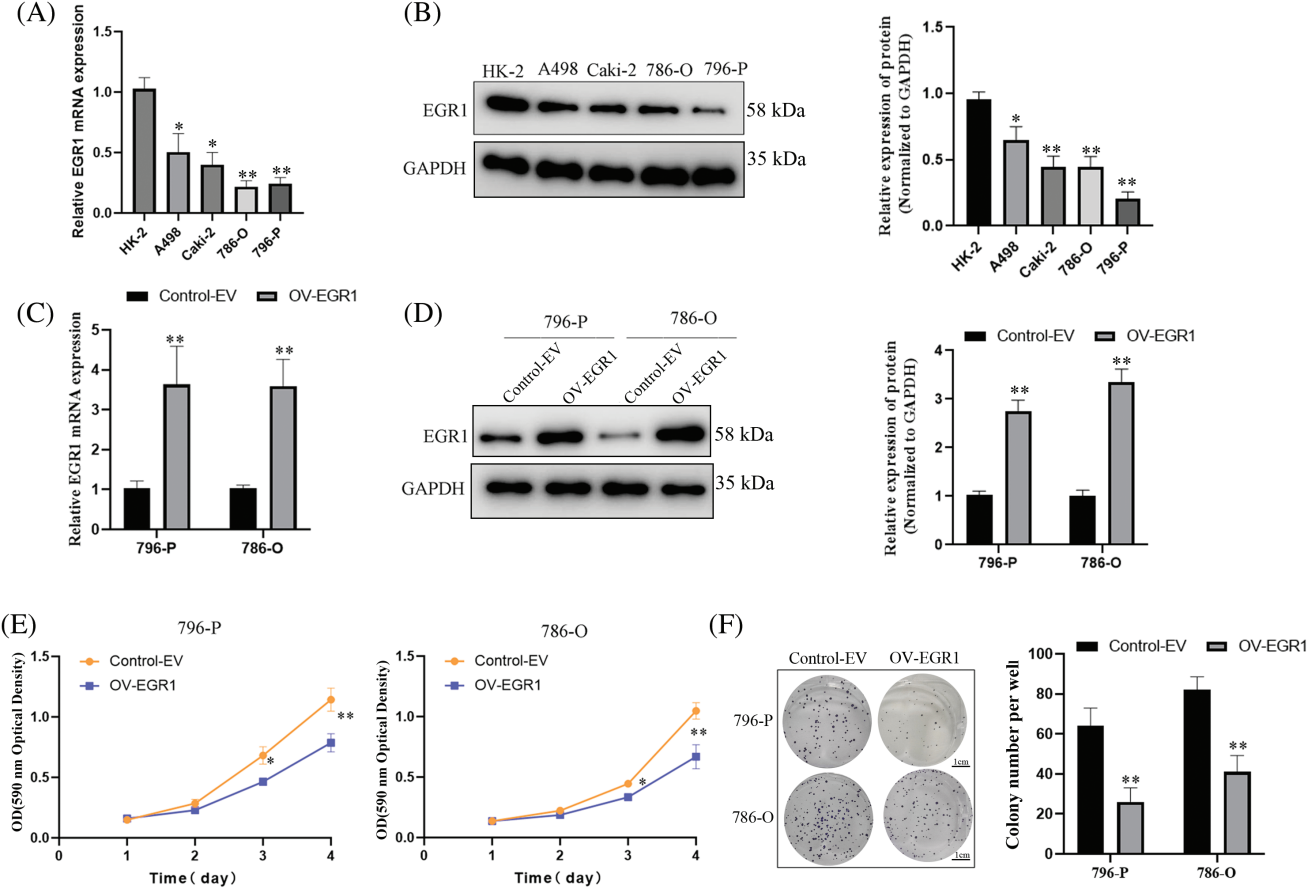
Effect of overexpressed EGR1 on ccRCC proliferation. Overexpression of EGR1 can inhibit ccRCC cell proliferation. (A, B) The EGR1 protein and mRNA expression in ccRCC and HK-2 cells. (C, D) The EGR1 protein and mRNA expression in transfected cells and blank Control (Control-EV). (E) The effect of overexpression EGR1 on ccRCC (786-O and 796-P) cells; and (F) The impact of overexpressed EGR1 on ccRCC (786-O and 796-P) cells colony formation. *p < 0.05, **p < 0.01.

The EGR1 expression in ccRCC cell lines (A498, 786-O, 796-P, ACHN) and renal tubular epithelial cells (HK-2) was assessed using fluorescence qPCR and WB analysis. These findings demonstrated that the EGR1 levels in the ccRCC cell lines (A498, 786-O, 796-P, and ACHN) were significantly lower (Fig. 3A,B). In order to further elucidate the function of EGR1 in ccRCC cells, OV-EGR1 and blank Control (control-EV) were synthesized and transfected into 796-P and 786-O cells. Analysis of gene and protein expression revealed that following transfection with the OV-EGR1, the level of EGR1 in 796-P and 786-O cells was higher than in the control-EV (Fig. 3C,D) (*p* < 0.01). The MTT assay results showed that the OV-EGR1 prevented the growth of ccRCC cells than the control-EV group (Fig. 3E) (*p* < 0.05). Furthermore, OV-EGR1 substantially reduced colony counts compared to the control-EV group (Fig. 3F).

### EGR1 overexpression prevented ccRCC cells from migrating and invading

**Figure 4:**
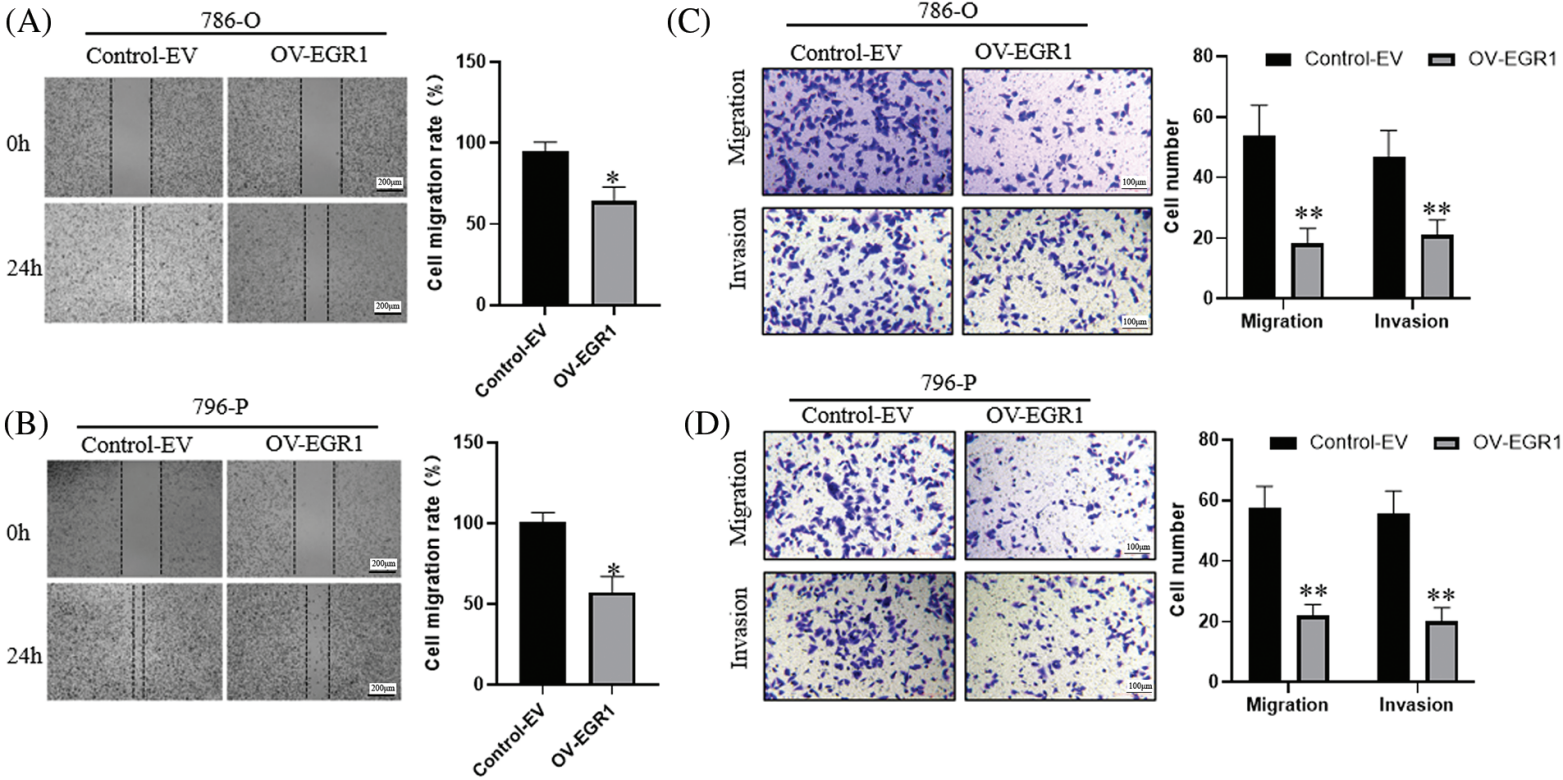
Inhibitory Effect of Overexpression of EGR1 invasion and migration of ccRCC cells. (A–B) Overexpression of EGR1 can inhibit ccRCC cells (786-O and 796-P) migration, (C, D) Overexpression of EGR1 could inhibit ccRCC (786-O and 796-P) cells migration and invasion. *p < 0.05, **p < 0.01.

Initially, a wound-healing experiment was carried out for ascertaining the effect of EGR1 on ccRCC cell motility. The results of Fig. 4A,B demonstrate that cells that overexpressed EGR1 had much less motility than the control group (*p* < 0.05). The transwell assay showed that, in comparison to the Control-EV group, EGR1 overexpression significantly decreased the migration and invasion of ccRCC cells (*p* < 0.01) (Fig. 4C,D).

### EGR1 affects MAPK15 signaling in ccRCC cells

**Figure 5:**
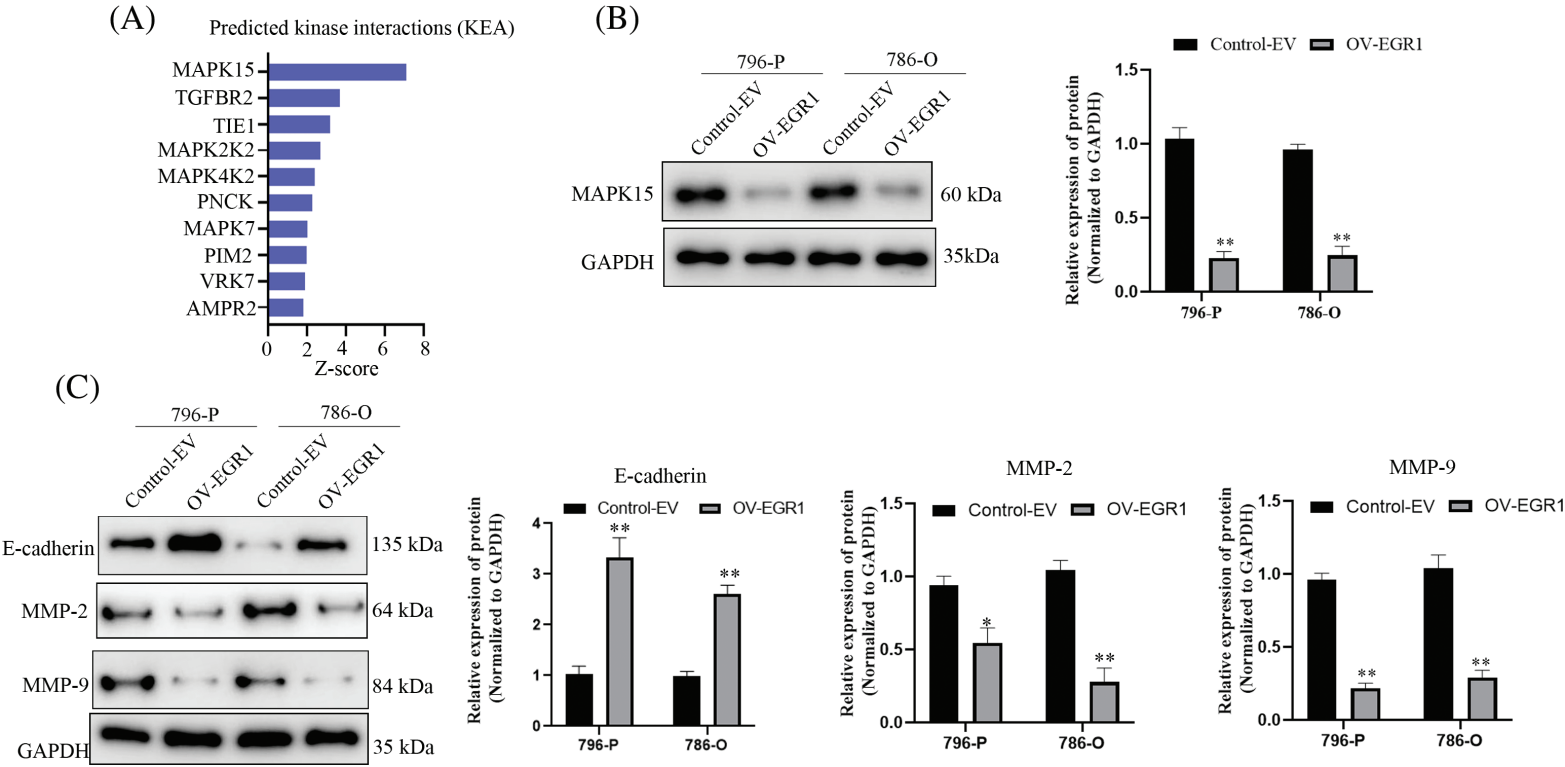
EGR1 affects MAPK15 signaling in ccRCC cells. Figure represents (A) Online Harmonizome (https://maayanlab.cloud/archs4/gene/EGR1#correlation) (accessed on 11 October 2024) predicts kinases that may be affected by EGR1. (B) WB analysis showing the impact of overexpression of EG1 on MAPK15 expression. (C) The effect of overexpression of EG1 on E-cadherin, MMP-9 and MMP-2 expression. *p < 0.05, **p < 0.01.

Kinase interactions (KEA) with EGR1 were predicted using the online Harmonizome website (https://maayanlab.cloud/archs4/gene/EGR1#correlation, accessed on 11 October 2024) to delve deeper into the molecular mechanism of EGR1 influencing ccRCC cell proliferation, invasion, and migration. The findings indicated a significant relationship between MAPK15 and EGR1 expression, as seen in Fig. 5A. The WB analysis showed that, compared to the Control-EV group, the enhanced expression of EGR1 significantly inhibited the expression of MAPK15 in ccRCC cells (Fig. 5B) (*p* < 0.01). Previous studies have shown that MAPK15 influences tumour cells’ migratory and invasive capabilities via modulating the MMPs pathway [19,20]. Comparing the overexpressed EGR1 group to the Control group, WB analysis showed a significant increase in E-cadherin and decrease in MMP-2 and MMP-9 expression (Fig. 5C) (*p* < 0.01). The results indicate that EGR1 affects the ccRCC cell migration and development through activation of MAPK15.

### Overexpression of EGR1 inhibited the ccRCC cells’ growth in vivo

**Figure 6:**
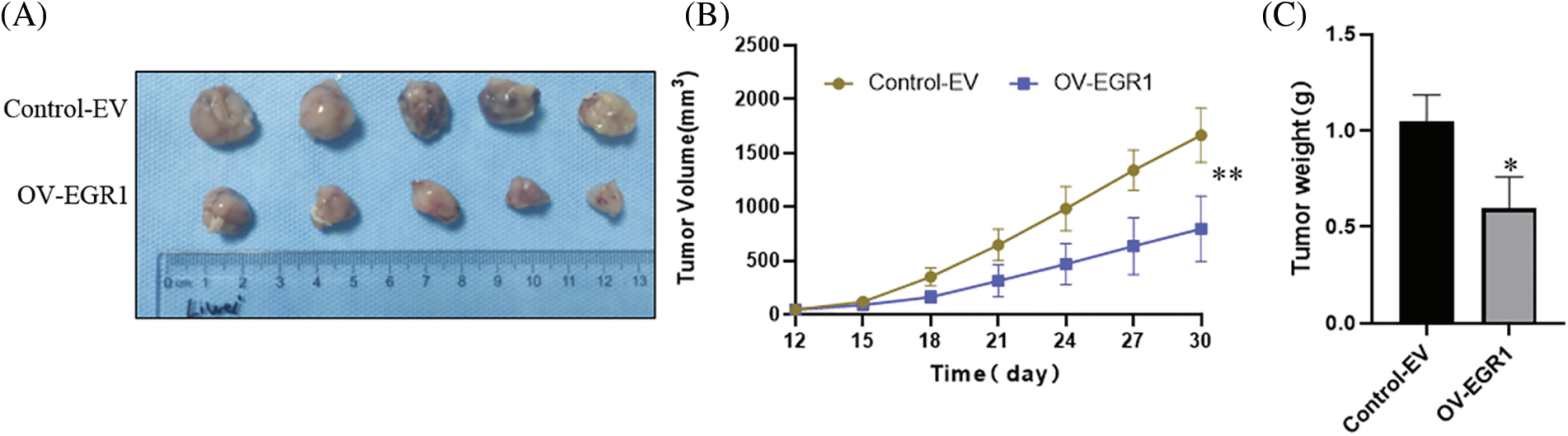
Effect of Overexpression of EGR1 on inhibition of ccRCC cell growth *in vivo*. (A) The representative tumor formation images of each group after 30 days, (B) Measuring tumor volume every three days until thirty days. (C) The tumor weight determined thirty days later. *p < 0.05, **p < 0.01.

To further demonstrate that EGR1 affects ccRCC cell proliferation *in vivo*. The subcutaneous tumor xenograft experiment performed with nude mice showed that tumors derived from cells with elevated EGR1 expression had a significantly reduced volume compared to those formed from control cells (Fig. 6A,B). Moreover, substantially lower tumor weight was observed in the OV-EGR1 tumor group (Fig. 6C) (*p* < 0.01). Therefore, Overexpression of EGR1 can suppress ccRCC progression by inhibiting malignant proliferation.

## Discussion

A recent study has indicated that EGR1, a key EGR family member, is crucial to cancer development and spread. It may also be participating in tumour cell invasion, angiogenesis, metastasis, and proliferation [7]. Nonetheless, EGR1 plays various roles in malignancies, such as promoting or inhibiting tumors. For instance, EGR1 increased pancreatic cancer’s liver metastasis through the P300/SNAI2 pathway [21]. Elevated EGR1 expression enhanced proliferation, invasion, and stemness by upregulating SOX9 in uterine cervical cancer cells [22]. In certain cancers, including breast cancer and hepatocellular carcinoma, EGR1 has been found to inhibit cancer cell growth and migration [23,24]. It is unknown if EGR1 promotes or suppresses tumor growth in ccRCC. These results showed that the expression of EGR1 was reduced in both ccRCC tissues and cells. Functional experiments additionally revealed that colony formation, cell motility, and invasion were all inhibited by ectopic EGR1 expression. Surprisingly, EGR1 can promote E-cadherin expression and inhibit MMP-9and MAPK15 expression levels.

Depending on the kind of cancer, EGR1 has been shown to have both tumor-promoting and tumor-suppressive properties. For instance, EGR1 has been documented to suppress migration and proliferation in breast cancer and hepatocellular carcinoma cells [23,24]. Conversely, in other cancers, EGR1 can enhance stemness and predict poor outcomes in uterine cervical cancer by promoting SOX9 expression [22]. These contrasting effects may be attributed to tumor heterogeneity or variations in genetic backgrounds. Tumor cells and the surrounding environment are influenced by tumor oncogenes in different ways, making the diversity among cancer cell populations an essential challenge in oncology. This observation might be attributed to tissue and cell-specific factors. EGR1 expression in ccRCC was assessed using an IHC assay to investigate further. According to the findings, EGR1 was strongly suppressed in ccRCC tissues. It was strongly correlated with the degree of differentiation, poor prognosis, metastasis of lymph nodes, and TNM stage for ccRCC patients. These results imply that EGR1 might function as a stand-alone ccRCC prognostic marker. Functional assays also indicated that excessive EGR1 expression reduced cell colony formation, stemness, and invasion. These results imply that EGR1 is a tumor suppressor that regulates ccRCC proliferation, invasion, and stemness.

Previous research has also indicated that EGR1 is crucial in various signaling pathways. In the cancer cells of the bladder, EGR1 promoted cell migration, invasion, and gemcitabine resistance by regulating SOX5 [25]. EGR1 has been shown to accelerate prostate cancer metastasis via the PI3K/PTEN/Akt pathway [26]. According to the current data, there is a substantial relationship between MAPK15 and EGR1 expression, with an apparent decrease in MAPK15 levels following EGR1 overexpression. MAPK15, the latest identified member of the MAPK family, is recognized for its upregulation across various cancer types [27]. It has been discovered that MAPK15 overexpression, an oncogene, stimulates the growth of gastric cancer cells by maintaining c-Jun expression [28]. MAPK15 is associated with lymph node metastasis and stimulates lung adenocarcinoma cell movement through prostaglandin E2 receptor and NF-κB p50 signaling [29]. The MAPK15 also promotes cell proliferation and protects against DNA damage in male germ cell tumors [30]. The current findings, which align with prior reports, show that MAPK15 is inhibited by exogenous expression of EGR1, which reduces ccRCC cell proliferation, migration, and invasion. Evidence from previous studies suggests that MAPK15 facilitates osteosarcoma invasion cell migration via modulation of MMP-9 expression [19]. MAPK15 regulates initial ciliogenesis in medulloblastoma cells, controlling hedgehog signaling [31]. The present findings also show that the increased expression of EGR1 effectively suppressed MMP-9/2 expression and promoted E-cadherin expression.

There are still limitations of this study. First, the present study revealed that overexpressing EGR1 diminished the MAPK15 expression level. Nevertheless, the precise methods by which EGR1 inhibits MAPK15 expression remain unclear. It is commonly recognized that EGR1 functions primarily as a transcription factor by binding to appropriate binding sites found in the regulatory regions of several genes [12,32]. So, in the following study, we will further explore using different experimental methods (such as chromatin immunoprecipitation and luciferase reporter gene). Secondly, this study identified EGR1 as an anti-metastatic gene in ccRCC cancer cells, showing its ability to inhibit cell migration and invasion. Although downstream targets such as E-cadherin and MMP-9/2 were examined, the analysis was insufficient. This study did not include other critical factors involved in cell migration and invasion, including N-cadherin, vimentin, snail, and twist.

## Conclusion

In conclusion, EGR1 is a potential prognostic biomarker for ccRCC. Elevated levels of EGR1 reduce cell proliferation, invasion, and stemness by downregulating MAPK15 expression. As a result, the results imply that focusing on the EGR1-MAPK15 pathway may offer a unique treatment strategy for ccRCC.

## Data Availability

The datasets generated during and/or analyzed during the current study are available from the corresponding author on reasonable request.
